# Antimicrobial and Methicillin Resistance Pattern of Potential Mastitis-Inducing *Staphylococcus aureus* and Coagulase-Negative Staphylococci Isolates from the Mammary Secretion of Dairy Goats

**DOI:** 10.3390/biology11111591

**Published:** 2022-10-29

**Authors:** Aikaterini Nelli, Chrysoula (Chrysa) Voidarou, Brigkita Venardou, Konstantina Fotou, Anastasios Tsinas, Eleftherios Bonos, George C. Fthenakis, Ioannis Skoufos, Athina Tzora

**Affiliations:** 1 Laboratory of Animal Health, Food Hygiene and Quality, Department of Agriculture, School of Agriculture, University of Ioannina, 47100 Arta, Greece; 2 Veterinary Faculty, University of Thessaly, 43100 Karditsa, Greece

**Keywords:** antimicrobial resistance, staphylococci, goat mastitis, MALDI-TOF MS, *mec* genes, caprine, intramammary infection

## Abstract

**Simple Summary:**

*Staphylococcus* spp. constitutes an important pathogenic microbe in goat farming due to its association with udder infection that results in decreased milk production and/or quality. This pathogen can be resistant to antibiotics as well as infect humans, thus, posing a public health concern. This study investigated the percentage and composition of staphylococci that are present in goat milk and evaluated the resistance pattern by testing different antibiotics. A further focus was given on the presence of resistance to a critically important antibiotic, namely methicillin. *Staphylococcus* spp. isolates were recovered from 45.9% of the milk samples which were differentiated into two groups, coagulase-negative staphylococci (CoNS, 72.3%) and *Staphylococcus aureus* (27.7%). Both groups were most commonly resistant to penicillins. CoNS exhibited resistance to a broader range of antibiotics than *S. aureus* with resistant CoNS being in higher number as well. Of the nine *S. aureus* and CoNS suspected of methicillin resistance, eight carried the *mecA* gene which is linked to this type of antibiotic resistance. The observed presence of resistance to antibiotics, especially methicillin, in staphylococci isolated from goat milk poses a concern for animal and human health.

**Abstract:**

*Staphylococcus* spp. is an important mastitis-inducing zoonotic pathogen in goats and is associated with antimicrobial resistance (AMR). The objectives of this study were to determine the prevalence and composition of staphylococci in individual mammary secretion (MS) samples of clinically healthy goats and to evaluate the phenotypic AMR pattern and the presence of methicillin resistance in the *Staphylococcus* spp. strains. *Staphylococcus* spp. isolates (*n* = 101) from the MS samples (*n* = 220) were identified to species level using matrix-assisted laser desorption/ionization time-of-flight mass spectrometry. The antimicrobial susceptibility testing included a disk diffusion assay and the determination of the minimum inhibitory concentrations (MIC) of resistant strains (*n* = 46). Presumptive methicillin-resistant strains (*n* = 9) were assessed for the presence of *mecA*, *mecC* and *SCCmec*/*orfx* genes. *Staphylococcus* spp. isolates were recovered from 45.9% of the MS samples, of which, 72.3% was identified as coagulase-negative staphylococci (CoNS), with the remaining being *Staphylococcus aureus*. CoNS and *S. aureus* were most commonly resistant to ampicillin (56.2% and 57.1%, respectively), penicillin (26.0% and 39.3%, respectively), amoxicillin (26 % and 25 %, respectively) and cephalexin (12.3% and 25%, respectively) in the disk diffusion method. CoNS exhibited a broader AMR pattern and a higher percentage of resistant strains than *S. aureus* in the disk diffusion and MIC methods. Of the nine oxacillin- and cefoxitin-resistant strains, three *S. aureus* and five CoNS strains carried the *mecA* gene and, thus, were identified as methicillin-resistant. The *mecC* gene was not found in any of the studied strains. The presence of AMR and methicillin resistance in caprine *S. aureus* and CoNS poses a concern for animal and public health.

## 1. Introduction

Clinical and subclinical mastitis are of principal importance in dairy goat herds due to their negative impact on the hygiene, quantity and quality of goat milk, thus affecting its availability for human consumption [1,2,3,4]. Subclinical mastitis has a higher incidence (5–30% or higher) than the clinical form (<5%) in dairy goats, with *Staphylococcus* spp. being the predominant causative agent in both cases, although other pathogens can also be involved [1,5,6,7]. In particular, coagulase-negative staphylococci (CoNS), namely *Staphylococcus caprae*, *Staphylococcus epidermidis*, *Staphylococcus xylosus*, *Staphylococcus chromogenes* and *Staphylococcus simulans*, most commonly lead to a subclinical infection and less frequently to a clinical disease, whereas the opposite is observed for *Staphylococcus aureus* [8,9].

The important role of *Staphylococcus* spp. as a mastitis-associated pathogen is also well-established in the indigenous goat herds of Greece. The first investigation of the situation in Greek farms was published by Kalogridou-Vassiliadou [10] in 1991, in which, staphylococci dominated among the isolated bacterial species (59.1%), with CoNS (56.9%) being the main representative followed by *S. aureus* (18%), other coagulase-positive staphylococci (CoPS, 22%) and unidentified *Staphylococcus* spp. (3.1%). The animals included in that study were considered clinically healthy; however, only isolates that were repeatedly recovered from consecutive milk samples of the same animal were reported, indicating that the presence of subclinical mastitis is quite likely [10]. Similarly, a more recent study on the causative agents of subclinical mastitis in dairy goats reared in Greek farms reported CoNS (50.2%) as the main causative agent followed by CoPS (34.5%), while also determining the incidence of subclinical mastitis at 24.1% and 31.7% in two successive years [11]. Of the CoPS, *S. aureus* represents the majority of the isolates, with a prevalence of 29% in lactating goats with subclinical mastitis, while this pathogen is also involved in cases of clinical mastitis [12,13]. 

*S. aureus* and CoNS have been associated with antimicrobial resistance (AMR), which leads to a reduced effectiveness of the available antimicrobials for treating infections caused by these pathogens. A major public health concern is the emergence of methicillin resistance in staphylococci worldwide [14]. Taking into account the routine use of β-lactams as mastitis treatment in dairy goats, this practice may facilitate the emergence of methicillin-resistant staphylococci in the produced milk and dairy products [15]. In several studies, *S. aureus* and CoNS strains resistant to one or more antimicrobials and even multi-drug resistant (MDR) strains have been isolated from goat milk collected from individual animals with [13,16,17,18] or without mastitis [19,20], with a high frequency of resistance to various members of the β-lactams groups commonly observed. Concerning the presence of methicillin-resistant *S. aureus* (MRSA) in goats with clinical/subclinical mastitis, the prevalence of such isolates was generally considered very low based on the detection of *mecA* and *mecC* genes [12,13,16]. MRSA strains harboring the *mecA* gene have also been isolated from milk samples of clinically healthy goats [18]. Interestingly, methicillin-resistant CoNS (MR-CoNS) have been reported in the milk of goats with [17] or without mastitis [19], suggesting that this staphylococcal group should also be considered during the evaluation of methicillin resistance in this animal species. AMR presence in *S. aureus* and CoNS poses a threat for public health, as the dissemination of resistant bacteria to humans, either due to direct contact with carrier animals or via the consumption of contaminated food, may lead to difficult-to-treat infections and/or the spread of AMR genes to human pathogens [21]. 

A low AMR prevalence in mastitis-associated *S. aureus* isolates has previously been observed in Greek goat farms [13]. A similar trend for *S. aureus* was also reported recently [22], whereas a higher AMR level was found for CoNS, ranging from a moderate to low level of resistance to various antimicrobials. However, in the aforementioned study, milk samples were collected from bulk tanks rather than individual animals for bacterial isolation. To our knowledge, no study has evaluated the AMR situation in the staphylococcal population of milk from clinically healthy goats. Concerning the research carried out on methicillin resistance prevalence, this has solely focused on the identification of MRSA isolates in caprine mastitic milk [12,13], with information on CoNS lacking. 

The objectives of this study were: (1) to identify *Staphylococcus* spp. in individual mammary secretion (MS) samples of clinically healthy goats from different farms in Greece using conventional microbiological techniques combined with matrix-assisted laser desorption/ionization time-of-flight mass spectrometry (MALDI–TOF MS), (2) to evaluate the AMR of the *Staphylococcus* spp. strains phenotypically and (3) to determine the presence of *mecA* and *mecC* genes in the presumptive methicillin-resistant isolates using real-time polymerase chain reaction (PCR).

## 2. Materials and Methods

### 2.1. Sample Collection

A total of 220 MS samples of 10–15 mL were collected into a sterile container from 11 goat farms as described previously [23]. Each sample was obtained from both mammary glands of individual clinically healthy goats (Skopelos and indigenous Greek breed) that were selected randomly from the animals of each herd. Samples were stored in a portable refrigerator and transported to the research Laboratory of Animal Health, Food Hygiene and Quality of School of Agriculture of University of Ioannina for culturing and bacteriological investigation for the presence of *Staphylococcus* species within 12 h. The number of goats selected for sampling represented 5% of the animal population of the aforementioned farms as carried out previously [24].

### 2.2. Experimental Design

The analysis of the collected MS samples was carried out as follows. The 220 MS samples were processed using conventional microbiological techniques, with 101 MS samples being positive for typical *Staphylococcus* spp. colonies. A single isolate from each positive sample (*n* = 101) was identified at the species level by Microflex LT Matrix-assisted laser desorption/ionization time of flight mass spectrometry biotyper (MALDI-TOF MS, Bruker Daltonic, Bremen, Germany). All *Staphylococcus* spp. strains (*n* = 101) were evaluated for their susceptibility to various antimicrobials using the disk diffusion assay. Resistant strains (*n* = 46) were further assessed concerning their magnitude of resistance by determining their minimum inhibitory concentration (MIC) for a selection of antimicrobials using a mastitis-specific VITEK card in the VITEK 2 COMPACT system (BioMérieux, Marcy, L’Étoile, France). Finally, four and five presumptive MRSA and MR-CoNS strains (*n* = 9) based on the observed resistant phenotypes to oxacillin and cefoxitin, respectively, were assessed for the presence of *mecA*, *mecC* and *SCCmec*/*orfX* genes. Details on each step of the analysis are provided in the subsequent sections.

### 2.3. Bacterial Isolation

For the isolation of *Staphylococcus* species, 10 μL from each MS sample was plated onto Columbia 5% defibrinated sheep blood agar (Oxoid, Basingstoke, UK) and incubated aerobically at 37 °C up to 72 h [25,26]. Morphologically distinct typical colonies of *Staphylococcus* spp. were purified by streaking across a second Columbia 5% defibrinated sheep blood agar plate to obtain single colonies of each isolate for the subsequent identification to species level.

### 2.4. Identification of Bacterial Isolates

The isolated bacteria were identified by MALDI-TOF MS as described previously [27,28,29]. The direct colony method, which involves the application of bacterial colonies from growth plates directly onto the steel plate before testing by MALDI-TOF MS, was used. The target plates were loaded into the Microflex LT instrument (Bruker Daltonic, Bremen, Germany), where a total of 240 laser shots in 40 shot steps were summarized, and each spot was measured twice automatically with AutoXecute acquisition control software (flexControl 3.4; Bruker Daltonic, Bremen, Germany). The generated peak list was matched against the database using the pattern-matching algorithm of MALDI Biotyper software version 3.4 (Bruker Daltonic, Bremen, Germany). A score of 2.0 or higher indicates high reliability at the species level and a score of 1.7 or higher but less than 2.0 indicates a match at the genus level. Scores below 1.7 are considered indicative of unidentified isolates [30]. Each run was internally calibrated using a bacterial test standard (BTS), which contains a typical *Escherichia coli* DH5 alpha peptide and protein profile plus additional proteins (Bruker Daltonic, Bremen, Germany).

### 2.5. Phenotypic Methods for Antimicrobial Resistance Detection in Staphylococcus species

#### 2.5.1. Disk Diffusion Method (Kirby–Bauer)

Antimicrobial sensitivity test (AST) of *Staphylococcus* spp. strains was carried out using the standard Kirby–Bauer disk diffusion method [31]. The following nine antimicrobial agents (μg/disk), commonly used in mastitis treatment, were tested: penicillin G (10), cephalexin CL (30), novobiocin NV (30), lincospectin LI (15-200), amoxicillin AMX (30), ampicillin AMP (30), chloramphenicol C (30), spectinomycin SH (25) and lincomycin MY (15) (Oxoid, Basingstoke, UK). Disk diffusion analysis was performed on Mueller–Hinton agar plates (Oxoid, Basingstoke, UK) with incubation conditions at 37 °C for 24 h. Quality control strains included *S. aureus* ATCC 33592 and *S. aureus* ATCC 25923. Inhibitory zone diameters were measured following incubation. The resistance breakpoints were interpreted according to the criteria provided by Clinical and Laboratory Standards Institute (CLSI) documents M100-S28 [32] and VET01S [33].

#### 2.5.2. Automated Commercial Phenotypic Method—VITEK 2 COMPACT System

Further phenotypic AST of the selected *Staphylococcus* spp. strains based on the disk diffusion assays was performed through the automated system VITEK 2 COMPACT (BioMérieux, Marcy, L’Étoile, France), which is one of the most commonly Food and Drug Administration (FDA)-approved systems for automated AST used in diagnostics [34,35]. Its intended use is the automated quantitative or qualitative susceptibility testing of isolated colonies of the most clinically significant aerobic bacteria and yeast. The VITEK 2 AST-GP79 Card Test Kit (BioMérieux, Marcy, L’Étoile, France) specific for the evaluation of the antimicrobial susceptibility of mastitis-associated *Staphylococcus* spp., which covers livestock-associated antimicrobials, was used. This system is based on the MIC technique reported by MacLowry and Marsh [36] and Gerlach [37], with the MIC values for each antimicrobial contained on the card determined at the end of the incubation cycle. The MIC results determine a category interpretation according to the interpretations defined by the FDA and CLSI. The AST-GP79 card included a total of 21 antimicrobials, of which, the following were tested: amikacin, benzylpenicillin, cefoxitin screen, cefquinome, ceftiofur, clindamycin, enrofloxacin, erythromycin, florfenicol, gentamicin, inducible clindamycin resistance, kanamycin, neomycin, oxacillin, tetracycline, tilmicosin, trimethoprim/sulfamethoxazole, tylosin, ampicillin, cefalotin and streptomycin high level (synergy). Ampicillin and streptomycin high level (synergy) were excluded from this analysis, as these antimicrobials have already been tested in the disc diffusion method. Regarding the cefalotin susceptibility analysis, inconsistent results were obtained for the different *Staphylococcus* spp. isolates and were thus excluded from this study. *S. aureus* ATCC 33592 (methicillin-resistant) and *S. aureus* ATCC 25923 (methicillin-susceptible) were used as the quality control strains. The tested strains were categorized following evaluation through the Advanced Expert System (AESTM) by the VITEK 2 COMPACT as susceptible, intermediate or resistant to the tested antimicrobials. Thus, the magnitude of resistance of each strain to a specific antimicrobial was expressed as the ratio of the MIC of the resistant strain and the MIC of a sensitive strain of the same *Staphylococcus* species.

### 2.6. Detection of mecA and mecC genes in Presumptive Methicillin-Resistant Strains

#### 2.6.1. DNA Extraction

Pure cultures of presumptive methicillin-resistant *Staphylococcus* spp. strains based on the AST results were grown in Columbia 5% defibrinated sheep blood agar plates at 37 °C overnight, and bacterial DNA was extracted using the DNeasy^®^ UltraClean^®^ Microbial Kit (Qiagen Inc., Toronto, ON, Canada) according to the manufacturer’s instructions. Double-stranded DNA concentration was determined by a Qubit fluorometer (Life Technologies, Nebraska City, NE, USA). The extracted DNA of each strain was stored at −20 °C until use.

#### 2.6.2. Real-Time PCR Reactions

Two different real-time PCR assays were applied for the detection of methicillin-resistant isolates. 

*Multiplex real-time PCR assay (1st real-time PCR assay*). This assay included a comprehensive amplification of three specific loci, the *nuc* gene, which is specific for *Staphylococcus* genus, the *SCCmec*/*orfX* junction and the resistance genes *mecA/mecC* combined using the methicillin-resistant PCR kit (Geneproof, Brno, Czech Republic). This kit provides qualitative detection by using three fluorophores: TexRed for the *nuc* gene presence, FAM for the *SCCmec*/*orfX* junction presence and Cy5 for the *mecA*/*mecC* presence. All of the steps for the PCR setup and the amplification procedure were carried out according to the manufacturer’s instructions.

*Real-time PCR assay (2nd real-time PCR assay)*. This assay was performed to detect *mecA* and *mecC* genes presence separately in each *Staphylococcus aureus* and CoNS strain. The sequences of the primers are listed in Table 1 as previously published by Stegger et al. [38]. The final reaction volume was 20 µL, containing 3 μL of template DNA, 3 μL (0.7 μM) of each primer, 10 μL 1X QuantiNova SYBR Green PCR Master Mix (Qiagen, Hilden, Germany) and 1 μL RNase-free water. Amplification was performed in a CFX96TM Real-Time PCR thermocycler (Biorad, CA, USA) using a denaturation step of 95 °C for 5 min followed by 35 cycles of 95 °C for 5 s and 60 °C for 30 s.

## 3. Results

### 3.1. Staphylococcus *spp.* Isolation and Species Identification by MALDI-TOF MS

Of the 220 MS samples processed, 101 were positive for typical *Staphylococcus* spp. colonies (45.9%), with a single isolate obtained from each MS sample. Analysis of the 101 isolates by MALDI-TOF MS led to the identification of 73 CoNS (72.3%) and 28 *S. aureus* (27.7%) strains, with a score range of 2.00 to 2.40. The CoNS strains were identified as follows: 17 *S. epidermidis* (23.3%), 16 *S. caprae* (21.9%), 8 *S. chromogenes* (11%), 8 *S. simulans* (11%), 6 *S. equorum* (8.2%), 4 *S. petrasii* (5.5%), 3 *S. pasteuri* (4.1%), 2 *S. capitis* (2.7%), 2 *S. warneri* (2.7%), 2 *S. lentus* (2.7%), 2 *S. haemolyticus* (2.7%), 1 S. *xylosus* (1.4%), 1 *S. hominis* (1.4%) and 1 *S. microti* (1.4%). 

### 3.2. Antimicrobial Susceptibility of S. aureus and CoNS Strains

In total, 73.3% of all *Staphylococcus* spp. strains (*n* = 74) were found to be resistant to at least one antimicrobial in the disk diffusion assay. Furthermore, nine strains (8.9%) were MDR, exhibiting resistance to three or more antimicrobial classes. At the species level, eighteen of twenty-eight *S. aureus* (64.3%) and fifty-six out of seventy-three CoNS strains (76.7%) were resistant to at least one of the antimicrobials. 

**Figure 1:**
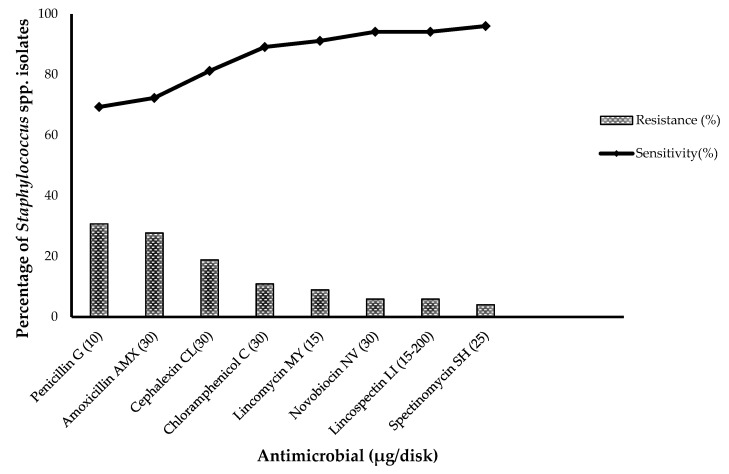
Susceptibility and resistance pattern of *Staphylococcus* spp. towards antimicrobials in the disk diffusion assay.

**Figure 2:**
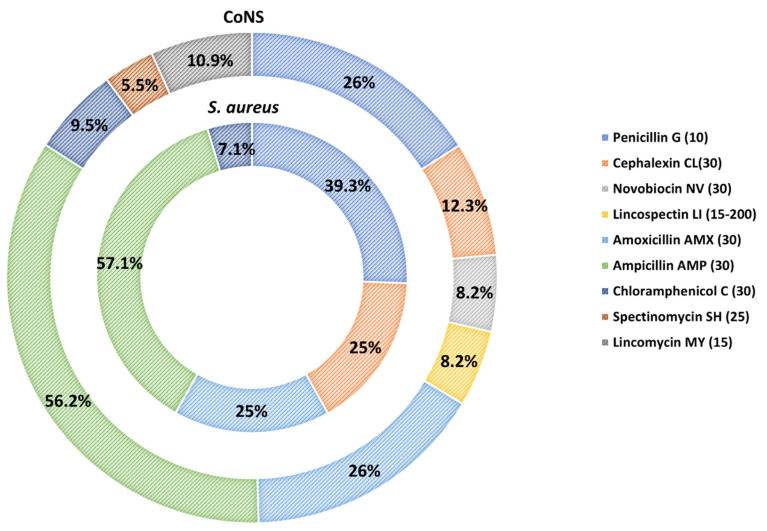
Resistance pattern of the *S. aureus* and CoNS strains to specific antimicrobials in the disk diffusion assay.

The resistance pattern to each antimicrobial for *Staphylococcus* spp. is presented in Figure 1, whereas the percentages of resistance for *S. aureus* and CoNS strains are shown in Figure 2. Resistance was most frequently observed against ampicillin (57.1% for *S. aureus* and 56.2% for CoNS, respectively) and penicillin (39.3% for *S. aureus* and 26.0% for CoNS, respectively), followed by amoxicillin (25 % for *S. aureus* and 26 % for CoNS, respectively) and cephalexin (25% for *S. aureus* and 12.3% for CoNS, respectively). A low level of resistance was observed against chloramphenicol (7.1% for *S. aureus* and 9.5% for CoNS, respectively). Interestingly, CoNS solely exhibited resistance to lincomycin (10.9%), novobiocin (8.2%), lincospectin (8.2%) and spectinomycin (5.5%).

### 3.3. MICs Determination of Resistant S. aureus and CoNS Strains

**Figure 3:**
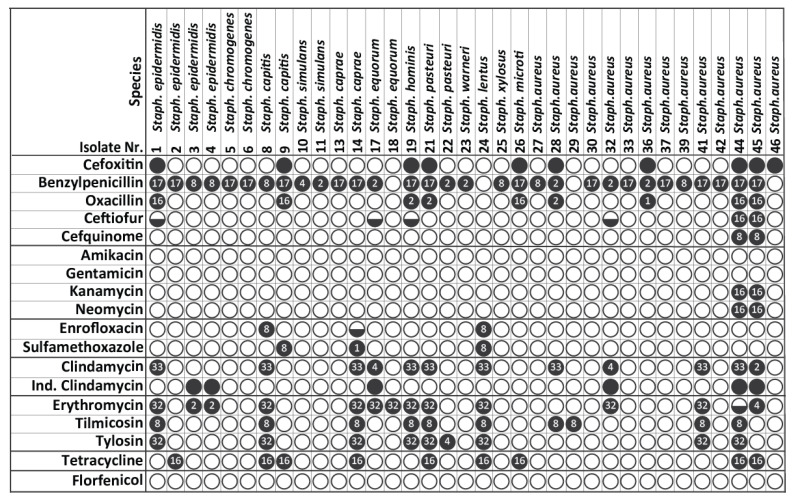
Determination of the antimicrobial resistance magnitude of *S. aureus* and CoNS strains (VITEK 2 COMPACT system); the black circle (●) indicates antimicrobial resistance and the number within the black circle indicates the magnitude of resistance expressed as the ratio of the MIC of the resistant strains and the MIC of the sensitive strains of the same *Staphylococcus* species, whereas the empty (o) and the half black circles () are used for susceptible and intermediate strains, respectively.

The MICs of the nine MDR *S. aureus* and CoNS strains, along with thirty-seven *S. aureus* and CoNS exhibiting resistance to several antimicrobials (*n* = 46) based on the disk diffusion assay, were determined for a selection of antimicrobials using the VITEK 2 COMPACT. The results for the strains exhibiting resistance to at least one antimicrobial are presented in Figure 3. Of the 46 isolates, 76.1% were resistant to at least one antimicrobial, whereas 23.9% were fully susceptible to all antimicrobials tested. The highest resistance was found to benzylpenicillin (88.6%), followed by erythromycin (37.1%), clindamycin (34.3%), tilmicosin (28.6%), cefoxitin (28.6%), oxacillin (25.7%), tylosin (25.7%) and tetracycline (25.7%). A low resistance ranging between 17.1–5.7% was observed for inducible clindamycin, trimethoprim/sulfamethoxazole, ceftiofur, cefquinome, kanamycin, neomycin and enrofloxacin, whereas no resistance was recorded for amikacin, gentamicin and florfenicol. 

A similar resistance pattern was also evident at species level for benzylpenicillin, erythromycin, clindamycin, tilmicosin, tylosin and tetracycline, with a higher resistance level consistently observed for CoNS. Resistance to ceftiofur, cefquinome, kanamycin and neomycin and to trimethoprim/sulfamethoxazole and enrofloxacin was solely observed for *S. aureus* and CoNS strains, respectively. In terms of presumptive methicillin resistance, four (28.6%) of the *S. aureus* strains and five (23.8%) of the CoNS strains were identified as possible MRSA and MR-CoNS, respectively, based on their phenotypic resistance to oxacillin and cefoxitin. Interestingly, two of the presumptive MRSA (44 and 45 *S. aureus—*Table 1) were the only strains associated with the observed ceftiofur, cefquinome, kanamycin and neomycin resistance.

### 3.4. Identification of MRSA and MR-CoNS via Detection of Methicillin Resistance-Associated Genes

The presumptive MRSA (*n* = 4) and MRCoNS (*n* = 5) strains were further investigated for the presence of *mecA* and *mecC* genes using real-time PCR. Eight of the nine strains carried the *mecA* gene, whereas none of the strains were positive for the *mecC* gene as presented in Table 2. As expected, all MRSA and MR-CoNS strains also carried the *nuc* and *SCCmec/orfX* junction genes (Table 2).

## 4. Discussion

A major issue in goat farming is the occurrence of subclinical mastitis due to the associated reduced milk quantity and quality and economic losses [9]. As *S. aureus* and CoNS are the predominant causative agents of mastitis [8,9], this study aimed to determine the presence and composition of staphylococci in the MS of clinically healthy goats from different herds in Greece. *Staphylococcus* spp. isolates were recovered from 45.9% of the 220 MS samples processed. Taking into consideration the findings of our previous study, in which, the staphylococci presence in MS samples was <1% in healthy goats based on the California mastitis test (CMT) and cytological examination [39], it is likely that those 45.9% of the clinically healthy goats included in the current study had subclinical mastitis. 

The identification of *Staphylococcus* spp. isolates at species level was carried out by MALDI-TOF MS, which has previously been used by our research group to identify clinical isolates [29], as well as by others to identify *S. aureus* and CoNS strains isolated from milk samples of goats with subclinical mastitis [12,19,40]. This easy-to-use, high-throughput and cost-effective technology provides rapid and accurate bacterial identification at genus and species level, rendering it a diagnostic tool with increasing popularity [41]. CoNS represented the majority of the staphylococcal isolates (72.3%) in agreement with previous studies in Greece [10,11], Italy, Brazil, Portugal and USA [16,17,19,20,40]. *S. epidermidis* (23.3%) and *S. caprae* (21.9%) were the dominant species, followed by *S. simulans*, *S. chromogenes* (11% each) and *S. equorum* (8.2%), with the remaining CoNS species being present at <5.5%. Most of the CoNS species identified in the current study have also been reported previously in goats with clinical or subclinical mastitis [10,16,17,19,20,40]. The variation in the prevalence of each species across studies is probably due to differences in geographic location, management practices and isolation and identification methodologies. *S. epidermidis*, *S. caprae*, *S simulans* and *S chromogenes* are important mastitis-inducing pathogens in goats associated with persistent infection, increased somatic cell counts and reduced milk yield [40,42,43,44]. *S. aureus* strains represented 27.7% of the staphylococci isolates, which was higher than the percentage reported elsewhere [16,17,19,20,40]. Despite the lower frequency of subclinical mastitis caused by *S. aureus*, its impact on somatic cells counts and milk yield is more severe compared to CoNS [11,43]. Thus, the presence of CoNS and *S. aureus* at high frequency (45.9%) in Greek goat farms indicates that there is a need for improvement in management and hygiene practices to achieve a better milk quantity and quality.

An interesting and unexpected finding was the isolation of *S. petrasii* (5.5%), a recently identified *Staphylococcus* species that consists of clinical isolates from humans and medical devices, except for a single strain that was recovered from a beer brewery air sample [45,46,47]. To our knowledge this is the first time that *S. petrasii* was reported in goats as well as in animals. As this *Staphylococcus* species has mostly been isolated from humans, it can be assumed that its presence in goats is the result of inadequate hygiene practices at milking. *S. microti* was another *Staphylococcus* species that has never been reported in goats; however, it has been previously identified as the causative agent of subclinical mastitis in dairy cows [48]. 

In the next part of this study, the AMR prevalence and pattern of the isolated *Staphylococcus* spp. strains were investigated. The initial AMR screening using the disk diffusion method showed that 64.3% of *S. aureus* and 76.7% of CoNS, representing 73.3% of all strains, were resistant to at least one antimicrobial. Similarly, a moderate-to-high AMR prevalence has previously been reported for *S. aureus* and CoNS strains isolated from goats with clinical [49] or subclinical mastitis [16,18,19], with a higher number of resistant CoNS than *S. aureus* strains in most cases. The findings of the current study disagree with those of earlier studies in Greek goat herds, in which, a lower AMR prevalence for *S. aureus* (2.3% and 20.1%) and CoNS (59.3%) was observed [13,22]. A probable explanation for this discrepancy is the fact that these studies used either a small number of caprine mastitis-associated *S. aureus* strains [13] or bulk-tank milk samples [22]. To further contribute to the understanding of the AMR situation in the staphylococci population in Greek goats, the current study included a larger sample size and individual MS samples to increase the accuracy of our findings and minimize the isolation of contaminant *Staphylococcus* spp. strains potentially originating from environmental sources (milking equipment, farm personnel) [50]. The AMR pattern for both *S. aureus* and CoNS strains included a high-to-moderate resistance to ampicillin, penicillin, amoxicillin and cephalexin, followed by a low level of resistance to chloramphenicol. The increased number of resistant *Staphylococcus* spp. strains to β-lactams is justified by the use of this group of antimicrobials as a routine mastitis treatment in Greek goat herds [15]. This observation further contradicts the earlier assumption that there is a low antimicrobial pressure in small ruminants in Greece using mastitis-associated *S. aureus* strains as an indicator [13]. An additional finding was the broader AMR pattern of CoNS (low resistance level to lincomycin, novobiotin, lincospectin and spectinomycin) compared to *S. aureus* as seen elsewhere [19,22], which indicates that these bacteria should also be considered when evaluating AMR prevalence in staphylococci. 

Based on the results of the disk diffusion assay, 46 *Staphylococcus* spp. strains (26 CoNS and 20 *S. aureus*) exhibiting phenotypic resistance to several of the tested antimicrobials were selected to determine their MICs for a wide range of different antimicrobials. The highest level of resistance for *S. aureus* and CoNS combined was observed for benzylpenicillin, followed by erythromycin, clindamycin, tilmicosin, cefoxitin, oxacillin, tylosin, tetracycline and inducible clindamycin, with several strains being MDR, whereas all strains were susceptible to amikacin, gentamicin and florfenicol. Resistance to penicillins, cephalosporins, macrolides, lincosamides and tetracyclines of *Staphylococcus* spp. strains isolated from goat milk samples has been reported, with variations among studies in the percentage of resistant strains to each antimicrobial class probably attributed to differences in the experimental design, AST method, tested antimicrobials and antimicrobial usage in the tested herds [17,19,22,49]. The presence of resistance to various antimicrobials renders the effective treatment of mastitis more challenging. Furthermore, *S. aureus* and CoNS are important human pathogens resulting in various clinical manifestations, such as foreign body-related, blood stream and skin and soft tissue infections, infective endocarditis, pneumonia and more [51,52]. Thus, the transmission of resistant strains from goats to humans via direct contact or the food chain may lead to difficult-to-treat infections or an exchange of AMR genes with the human staphylococci [53,54]. 

Another important finding was that resistance to most of the above-mentioned antimicrobials was consistently at a higher prevalence in CoNS compared to *S. aureus* strains, a trend similar to previous studies [19,20,22]. It is worth noting that CoNS are considered an important AMR gene reservoir that contributes to the intra- and inter-species dissemination of resistance within the *Staphylococcus* genus, with animal hosts and their food products likely acting as major AMR gene exchange hubs [19,54,55]. To our knowledge, this is the first study that has provided evidence on resistant CoNS directly isolated from the udder of goats in Greece. Taking all of the above together, a more in-depth investigation of AMR in this bacterial population in livestock including goats is required.

Of interest to this study was the identification of nine presumptive MRSA (*n* = 4) and MR-CoNS (*n* = 5) strains, as indicated by the observed resistance to oxacillin and cefoxitin. To determine the prevalence of methicillin resistance, these strains were evaluated for the presence of *mecA* and *mecC* genes and the *SCCmec*/*orfX* junction gene. The *mecA* and *mecC* genes, located on the staphylococcal cassette chromosome mec (*SCCmec*) mobile genetic element, provide resistance to β-lactams by encoding for an altered penicillin-binding protein (PBP2′ or PBP2α) characterized by a low affinity to this antimicrobial class [56]. All strains except for one *S. aureus* strain harbored the *SCCmec*/*orfX* junction and *mecA* genes and, thus, were identified as MRSA and MR-CoNS. None of these strains harbored the *mecC* gene. The results of the current study concerning the MRSA prevalence are in agreement with an earlier study [12] supporting the notion that this pathogen is present at a low frequency in the goat herds of Greece. However, this is the first report of MR-CoNS strains, namely *S. epidermidis*, *S. capitis*, *S. hominis*, *S. pasteuri* and *S. microti*, being isolated from goat herds in Greece. Previous studies have observed methicillin-resistant *S. epidermidis* and *S. hominis* in milk samples from goats with [17] or without mastitis [19], whereas methicillin-resistant *S. capitis* and *S. pasteuri* strains have been recovered from mastitic milk of dairy cows [57,58]. The presence of AMR and methicillin resistance in *S. microti*, has not been reported previously. This observation might be indicative of the occurrence of interspecies resistance dissemination, as an in silico investigation of the publicly available *S. microti* sequences revealed the absence of AMR genes in this *Staphylococcus* species [55]. This assumption is further supported by previous studies that showed the occurrence of AMR gene exchange between *Staphylococcus* species, including a caprine isolate under in vitro conditions [19,54] and a higher frequency of such events in vivo [53]. Future research should provide better insight toward the role of CoNS and MR-CoNS concerning the dissemination of resistance among staphylococci and the potential impact to animal and human health.

## 5. Conclusions

This study further confirmed the dominant presence of CoNS over *S. aureus* in the MS of goats and provided information regarding the CoNS composition by utilizing MALDI-TOF MS, an accurate and easy-to-use diagnostic tool for rapid bacterial identification. A high prevalence of phenotypic resistance in the *Staphylococcus spp.* strains was recorded to various antimicrobials, predominantly penicillins and cephalosporines, commonly used as mastitis treatment. Interestingly, the resistant CoNS strains were present at a consistently higher percentage than the resistant *S. aureus* strains. Furthermore, three MRSA strains and five MR- CoNS strains, isolated for the first time from caprine MS in Greece, were identified by the detection of the *mecA* gene. The observed AMR and methicillin resistance in the mastitis-associated CoNS and *S. aureus* strains may render the selection of an effective treatment challenging in the Greek goat herds, while additionally posing a threat to public health due to their zoonotic potential. Of particular concern is the likelihood of resistant CoNS acting as an AMR reservoir, which should be explored in future studies. 

## Figures and Tables

**Table 1:** PCR primers for *mecA* and *mecC* genes.

Target Gene	Nucleotide Sequence (5′-3′)	PCR Product Size (bp)
*mecA*	F: TCCAGATTACAACTTCACCAGG	162
	R: CCACTTCATATCTTGTAACG	
*mecC*	F: GAAAAAAAGGCTTAGAACGCCTC	138
	R: GAAGATCTTTTCCGTTTTCAGC	

**Table 2:** Presence of *nuc*, *SCCmec*/*orfX*, *mecA* and *mecC* in the tested MRSA and MR-CoNS strains.

Isolates	1st Real Time PCR Assay			2nd Real Time PCR Assay	
	*nuc*	*SCCmec*/*orfX*	*mecA*/*mecC*	*mecA*	*mecC*
1.CoNS *	+	+	+	+	−
9.CoNS *	+	+	+	+	−
19.CoNS *	+	+	+	+	−
21.CoNS *	+	+	+	+	−
26.CoNS *	+	+	+	+	−
28.SA *	+	+	+	+	−
36.SA *	+	−	−	−	−
44.SA *	+	+	+	+	−
45.SA *	+	+	+	+	−

* CoNS: coagulase-negative staphylococci, 1: *S. epidermidis*, 9: *S. capitis*, 19: *S. hominis*, 21: *S. pasteuri*, 26: *S. microti*, SA: *S. aureus* (28, 36, 44, 45). − absence; + presence.

## Data Availability

Data are contained within the article.
